# DNA metabarcoding identifies urban foraging patterns of oligolectic and polylectic cavity-nesting bees

**DOI:** 10.1007/s00442-022-05254-0

**Published:** 2022-09-13

**Authors:** Kristen Fernandes, Kit Prendergast, Philip W. Bateman, Benjamin J. Saunders, Mark Gibberd, Michael Bunce, Paul Nevill

**Affiliations:** 1 Trace and Environmental DNA (TrEnD) Laboratory, School of Molecular and Life Sciences, Curtin University, Bentley, WA 6102 Australia; 2 Section for Molecular Ecology and Evolution, Faculty of Health and Medical Sciences, Globe Institute, University of Copenhagen, Copenhagen K, Denmark; 3 Food Agility CRC Ltd, 175 Pitt St, Sydney, NSW 2000 Australia; 4 School of Molecular and Life Sciences, Curtin University, Bentley, WA 6102 Australia; 5 Behavioural Ecology Lab, School of Molecular and Life Sciences, Curtin University, Bentley, WA 6102 Australia; 6 Centre for Crop and Disease Management, School of Molecular and Life Sciences, Curtin University, Bentley, WA 6102 Australia; 7 The Institute of Environmental Science and Research (ESR), Kenepuru, Porirua, 5022 New Zealand; 8 MBioMe - Mine Site Biomonitoring using eDNA Research Group, Trace and Environmental DNA (TrEnD) Laboratory, School of Molecular and Life Sciences, Curtin University, Bentley, WA 6102 Australia

**Keywords:** Urbanisation, DNA metabarcoding, Native bees, Trap nests, Floral preferences

## Abstract

Urbanisation modifies natural landscapes resulting in built-up space that is covered by buildings or hard surfaces and managed green spaces that often substitute native plant species with exotics. Some native bee species have been able to adapt to urban environments, foraging and reproducing in these highly modified areas. However, little is known on how the foraging ecology of native bees is affected by urbanised environments, and whether impacts vary among species with different degrees of specialisation for pollen collection. Here, we aim to investigate the responses of native bee foraging behaviour to urbanisation, using DNA metabarcoding to identify the resources within nesting tubes. We targeted oligolectic (specialist) and polylectic (generalist) cavity-nesting bee species in residential gardens and remnant bushland habitats. We were able to identify 40 families, 50 genera, and 23 species of plants, including exotic species, from the contents of nesting tubes. Oligolectic bee species had higher diversity of plant pollen in their nesting tubes in residential gardens compared to bushland habitats, along with significantly different forage composition between the two habitats. This result implies a greater degree of forage flexibility for oligolectic bee species than previously thought. In contrast, the diversity and composition of plant forage in polylectic bee nesting tubes did not vary between the two habitat types. Our results suggest a complex response of cavity-nesting bees to urbanisation and support the need for additional research to understand how the shifts in foraging resources impact overall bee health.

**Supplementary Information:**

The online version contains supplementary material available at 10.1007/s00442-022-05254-0.

## Introduction

Within the next century, urban areas around the world will grow rapidly, with some models predicting that by 2100, the global area of urban land will increase to 5.9 times the area that it was in 2000, to cover over 3.6 million km^2^ of land (Gao and O’Neill 2020). Higher human population density and associated urbanisation can cause the loss of biodiversity and endemic species (McDonald et al. 2018), not only by clearing native vegetation, but by permanently modifying the natural landscape through the creation of built-up space (buildings, roads, and other structures) and managed green spaces (Harrison and Winfree 2015). Even in regions with high biodiversity, such as the southwest of Australia, urban green spaces have a generally higher level of plant diversity than remnant bushland, stemming from the increased planting of exotic species (Prendergast 2020). This pattern has also been observed in other areas of the globe, such as the United Kingdom (Davies et al. 2009). However, native species relying on the pollen and nectar resources from native plants may not always be able to access exotic floral resources in urbanised environments. This is because in regions with high endemism and species richness, ecosystem dependencies are common between groups of flora and fauna species (Johnson 2010). Additionally, there are many ornamental varieties of plants in residential gardens that offer minimal nectar or pollen rewards for insects (Corbet et al. 2001). Therefore, the clearing of native habitat in these ecosystems due to urbanisation can cause the destabilisation of dependant ecosystem networks, resulting in local extinctions and ecosystem functional collapse (Sánchez-Bayo and Wyckhuys 2019).

Overall, the impacts of urbanisation on organisms are highly varied, and how a species will respond is dependent on its ecological requirements, functional and life-history traits, the spatial scale of investigation, geographic region, and the intensity of urbanisation (Theodorou 2022). Species more at risk from urbanisation include specialist cavity-nesting birds, short-distance migrants, and narrowly distributed species (Luck and Smallbone 2010). Whilst increased degrees of urbanisation generally result in a decline in species diversity, paradoxically some urban areas become a refuge for native biodiversity (Goddard et al. 2010). For example, urban parks in San Francisco, USA, supported higher abundances of generalist native bumblebee (*Bombus spp.*) than parks outside of the urban area (McFrederick and LeBuhn, 2006). Additionally, populations of the European common brown frog (*Rana temporaria*) have shown increases in urban gardens and parks, whilst declining in rural areas (Carrier and Beebee 2003). For insects, urban areas have been found to benefit cavity-nesting, small-bodied, generalist, and exotic species (Buccholz and Egerer 2020; Fitch et al. 2019). Partially, this is due to the value of certain traits of urban gardens that can enhance the retention of biodiversity. The value of a particular urban garden for insects will depend on the built form, vegetation cover, vegetation composition, management procedures, interconnectivity with other green spaces, and human population density (Persson et al. 2020).

Native bees play a key role in functional ecosystems and maintaining their populations in urban areas is crucial. They perform pollinator services across the globe, for both crop and native plant species (Winfree et al. 2008). The survival of native bees in urban areas is dependent on species’ ecology and foraging preferences: in some regions, there is evidence of co-evolution of bee species with specific native flowering plants (Phillips et al. 2010; Menz et al. 2011), implying that the loss of certain plant groups can have profound impacts on resource availability for their associated visitors. The level of forage flexibility of individual bee species will determine whether: (a) the species can access a variety of floral resources (i.e., a generalist) or (b) whether they are restricted to a certain group of plants (i.e., a specialist). The loss of native flora can restrict the resource availability for specialist bee species to a narrower range of available flora (Prendergast and Ollerton 2021). For many native bee species, there can also be a preferential avoidance of exotic plant species (Buchholz and Kowarik 2019). Lecty refers to the degree of trophic specialisation for pollen collection (Cane and Sipes 2006). Bees that exhibit specialisation in their diets for pollen from a particular taxon are known as “oligolectic” bees; these bees are believed to be constrained to a narrow resource breadth by physiological, temporal, or environmental factors (Fox and Morrow 1981; Devictor et al. 2010). “Polylectic” bee species, however, can feed on a wide variety of pollen sources from different families of plants.

There is a current lack of available knowledge on floral specialisation for many bee species (Bogusch et al. 2020). To capture the full spectrum of floral resources used by bees requires a combination of foraging observations and pollen analysis from netted bees (Cane and Sipes 2006). Quantification of floral resource usage by many native bees has been largely based on observation data, rather than on pollen collection (Roulston and Cane 2000; Bosch et al. 2009). Additionally, there is evidence to suggest that lecty is a spectrum, rather than binary, and that resource usage can be varied based on sex or blooming phase of preferred flowering plants (Ritchie et al. 2016). For oligolectic bees, some species have been documented to access nutrition from nectar, floral oils, or pollen from less preferred plants where preferred host plants may be rare or have limited blooming periods (Wcislo and Cane 1996). However, there is still limited understanding of how less preferred forage resources can impact reproduction or overall bee health (Filipiak and Filipiak 2020). Therefore, if conservation actions are needed to protect native bee populations, it is important to understand the preferred foraging resources and the range of forage flexibility of native bees in an area under threat.

Artificial nesting blocks—‘trap nests’ or ‘bee hotels’—can be beneficial in understanding foraging behaviour of solitary cavity-nesting bees (MacIvor 2017; Staab et al. 2018) and pollen–bee and host–parasitoid interactions between cavity-nesting bee taxa and the surrounding environment (Krombein 1967). Within the cavities, female bees construct brood cells, which they provision with pollen and nectar and then lay an egg. Although not all bee species use trap nests, appropriately designed trap nests can allow for the detection of a wide diversity of bee species, including both males and females of the same species that may not otherwise be observed in field surveys (Prendergast et al. 2020). Additionally, as cavity-nesting bees are central place foragers, the species that use trap nests forage in an area around the nest that is limited by their flight range (Zurbuchen et al. 2010b). This means that cavity-nesting bees can be considered indicators that help understand changes in the local environment (Tscharntke et al. 1998). Studying the larval provisions (nectar and pollen) within trap nests can be a valuable tool to understand foraging resource availability within a season. Forage resource availability for solitary bees partly determines the number, size, and sex ratio of offspring (Pitts-Singer, 2015). This is because female bees can control the offspring sex and body size, and a shortage of resources can result in reduced maternal investment favouring the production of fewer young that require less resources, often males (Seidelmann et al. 2010). Therefore, studying the resources within trap nests can provide valuable information into the future health and functionality of changed ecosystems.

As morphological identification of plant materials requires expertise in taxonomic identification across multiple families of plants, genetic tools are being increasingly implemented to aid in pollen identification, primarily DNA metabarcoding (Pornon et al. 2016; Bell et al. 2017). The value of DNA metabarcoding is its ability to identify species accurately and rapidly, which in turn can reveal fine-scale interactions that may not be detected from the observation of pollinator–plant interactions alone (Pornon et al. 2016, 2017). This is especially useful in understanding the impacts of urbanisation on native bees, especially in regions where these species may be understudied. DNA metabarcoding works by (i) extracting DNA from environmental or bulk specimen samples, (ii) amplifying the DNA using nucleotide-labelled primers (Bohmann et al. 2022), and (iii) sequencing on high-throughput sequencing platforms and identifying the resulting sequences using reference sequence databases (Taberlet et al. 2012) or via taxon-independent approaches (e.g., OTUs). For taxonomic assessment of pollen, metabarcoding has allowed simultaneous identification of plant taxa across multiple species and samples (Taberlet et al. 2012). DNA metabarcoding has been used to identify the taxonomic constituents of pollen loads from pollinators (Pornon et al. 2016, 2017; Bell et al. 2017), brood cells within trap nests (Gresty et al. 2018; Voulgari‐Kokota and Ankenbrand 2019), honey (De Vere et al. 2017), and pollen traps at the entrances of beehives (Keller et al. 2015). To our knowledge, DNA metabarcoding has yet to be used to document foraging behaviour and preferences from cavity-nesting bee species in urban environments or in Australian ecosystems.

We used DNA metabarcoding of the biological material from trap nests to investigate how eight species of Australian oligolectic or polylectic cavity-nesting bees utilise forage resources in urban bushland remnants compared to residential gardens. Our hypothesis was that polylectic bees will gather a greater diversity of plant material in their trap nests compared to oligolectic bee species in both habitat types (residential gardens and bushland remnants). Furthermore, we predict that because of the greater floral diversity in residential gardens (Prendergast and Ollerton 2021), there will be a higher diversity and varied composition of plants collected by polylectic bee species in residential gardens compared to bushland remnants. We anticipate that in residential gardens, the forage composition within trap nests of oligolectic bees will not change due to their specialisation or be reduced, because only a subset of plant species will be present.

## Materials and methods

### Experimental design

**Fig. 1:**
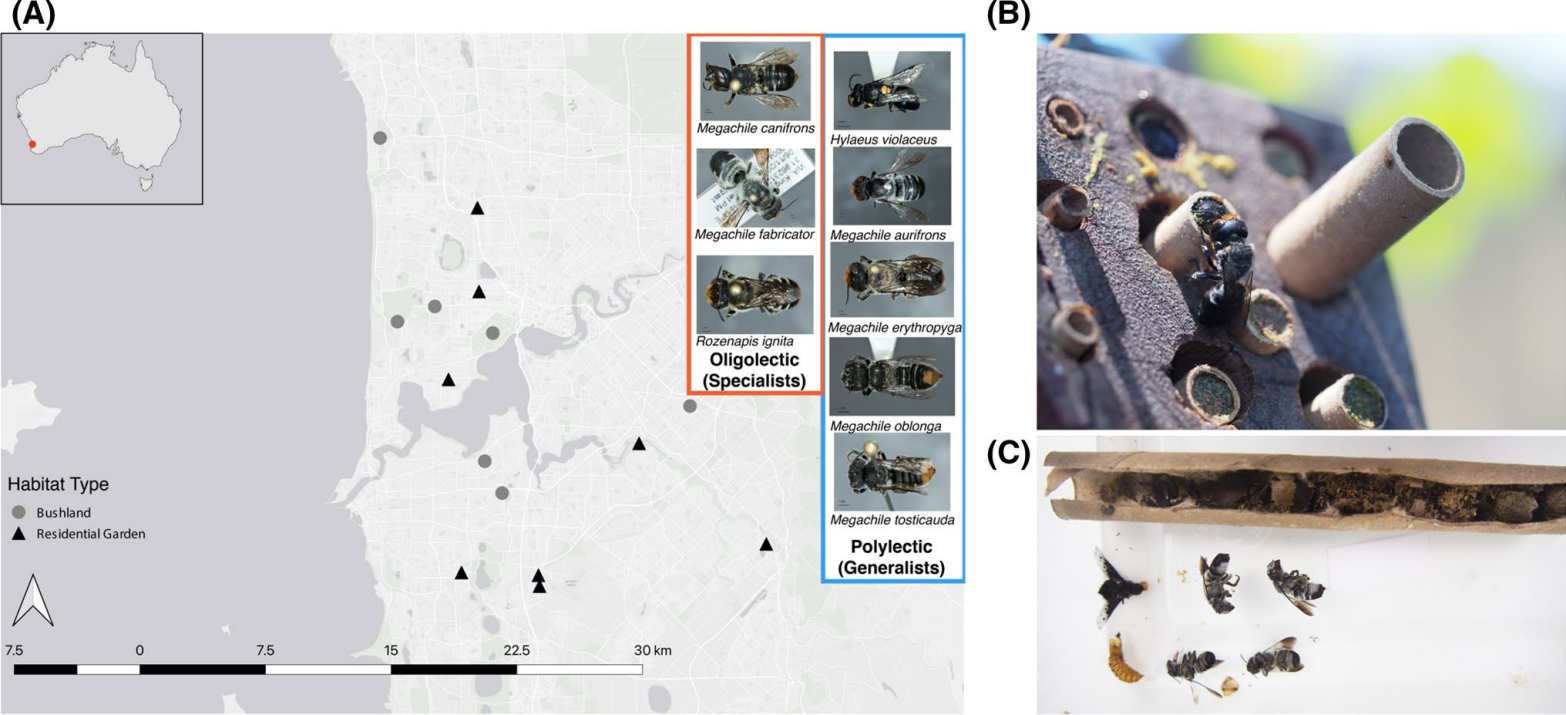
**A** Map of the study sites in Perth, Western Australia showing locations of bushland remnant (grey circle) and residential garden (black triangle) habitat types with images of bee species included in this study alongside. Species in the blue box are polylectic (generalists), whilst those in the orange box are oligolectic (specialists). Images of bees were taken by K.S. Prendergast using the WA Museum’s imaging microscope and stacking software. **B** Bee visitor to a trap nest. Image by K.S Prendergast. **C** Inside a *M. fabricator* nesting tube showing four mature adults, larvae, a parasitic bombyliid *Anthrax* sp. fly, and the remaining pollen and plant material debris. Images by K.S Prendergast

To investigate the impacts of urbanisation on oligolectic and polylectic native bee foraging behaviour, we collected nesting tubes from trap nests from 14 sites across the Perth metropolitan region, in southwest Australia (Fig. 1A). This region is a known biodiversity hotspot with high levels of species endemism and diversity, but it is under threat from various anthropogenic factors, such as urbanisation (Phillips et al. 2010). The 14 sites represented two habitat types: native bushland remnants and residential gardens, seven in each (Fig. 1A). Trap nests and the recorded habitat characteristics used in this study were sourced from a previous study investigating sampling methods for Western Australian native bees (Prendergast et al. 2020). From these trap nests, we examined eight species of native bee that had varying levels of diet specialisation (lecty). Oligolectic bees collect pollen from one plant family (specialists) and polylectic bees collect pollen from a greater diversity of plant families (generalists) (Cane and Sipes 2006). Our study included three specialist and five generalist bee species (Table S1). The oligolectic bee species were: *Megachile* (*Hackeriapis*) *canifrons* (Smith, 1853), *Megachile* (*Mitchellapis*) *fabricator* (Smith, 1868), and *Rozenapis ignita* (Smith, 1853). The polylectic bee species were: *Hylaeus* (*Euprosopis*) *violaceus* (Smith, 1853), *Megachile aurifrons* (Smith, 1853), *Megachile erythropyga* (Smith., 1853), *Megachile* (*Hackeriapis*) *oblonga* (Smith, 1879), and *Megachile* (*Hackeriapis*) *tosticauda* (Cockerell, 1912). Lecty for each species were designated based on observations of bees foraging on flowers across southwest WA from 2016 to 2021 by K. S. Prendergast (unpub), observations from Prendergast and Ollerton (2021), and, if present, records in Houston (2000). Where possible, equal numbers of nesting tubes were selected from each habitat type, this ranged from a minimum of 6 to a maximum of 10 nesting tubes for each bee species (Table S1). Habitat Characteristics and Native Bee Observations*.*

We used the following habitat information gathered from Prendergast et al. (2020) and Prendergast and Ollerton (2021) at each of the 14 sites to distinguish between remnant bushland and residential gardens: bare ground cover (a proxy for nesting space for ground-nesting bee species); the number of woody plants (a proxy for nesting material for cavity-nesting bee species); the total area of the site; percentage of built space; native floral species richness; the number of native flowers; and the proportion of native flowers to horticultural species both in richness and in number (for descriptions, see Table S2).

Floral hosts for each species were designated based on observations of bees foraging on flowers across southwest WA from 2016 to 2021 by K. S. Prendergast (unpub) and, if present, records in Houston (2000). Intertegular span (Cane 1987) was measured from dorsal stacked photos of a female of each species (Canon DLSR, 100 mm lens, 1:1 magnification, f-stop 8). The images were imported into Adobe Photoshop and measured using the set measurement scale and ruler features. Intertegular span is the distance between the points where the wings attach to the thorax. It has been used as an estimate of bee size and flight abilities (Cane 1987). Greater intertegular span is a proxy for greater potential foraging distance (Wright et al. 2015). The largest bee species in our study was the oligolectic *Megachile (Mitchellapis) fabricator,* and the smallest species was the polylectic *Hylaeus violaceus* (Table S1)*.*

### Sample processing

Once young bees had emerged from nesting tubes, each tube was separated by site and species, constituting a sample. In total, we sampled 148 nesting tubes. Where possible, equal numbers of nesting tubes were selected for each species from each habitat type (ranging from five to ten tubes per habitat type) (Table S1). Sterilised forceps were used for each sample to scrape the insides of nesting tubes of frass (larvae faecal matter), pollen and, for some species, resin debris (Fig. 1C). Scrapings were then homogenised using a PreCellLys 24 2.8 mm Ceramic Bead Kit and a Minilys Personal Homogeniser for 3 min at 5000 rpm (Bertin Instruments, France).

### DNA extraction, PCR amplification, and sequencing

DNA extraction was conducted using a DNeasy Plant Mini Kit on an automated Qiacube (Qiagen, The Netherlands) modified with a 450 µL starting volume of digest and a 100 µL elution volume. Negative extraction controls were included for every 48 samples (*n* = 4).

Two plant metabarcoding assays were used to analyse the bee nesting tube contents across two gene regions of varying lengths: a shorter assay of ~ 30–143 bp targeting the P6 loop of the chloroplast *trn*L (UAA) intron (primers g and h; Taberlet et al. 2007) and a longer ~ 563 bp ITS2 assay (ITS2_S2F/S3R; Chen et al. 2010). Quantitative PCR (qPCR) was carried out on all samples to assess the amplification efficiency and presence of PCR inhibitors using serial dilutions of undiluted, 1:10 and 1:100. qPCR reactions were carried out in 25 µl reactions containing: 1 U of AmpliTaq gold, 1 × PCR Gold Buffer and 2 mM MgCl2 (all from Applied Biosystems, USA), 0.4 mg/mL bovine serum albumin (Fisher Biotec, Australia), 0.25 mM dNTPs (Astral Scientific, Australia), 0.4 µM of each forward and reverse primer, 0.6 µL of 1/1000 SYBR Green (Invitrogen, USA), and 2 µL of template DNA. The qPCR conditions for *trn*L were as follows: 95 °C for 5 min, followed by 45 cycles of 95 °C for 30 s, 52 °C for 30 s, and 72 °C for 45 s, with a final elongation at 72 °C for 10 min. For ITS2, the qPCR conditions were as follows: 94 °C for 5 min, followed by 45 cycles of 94 °C for 30 s, 56 °C for 30 s, and 72 °C for 45 s, with a final elongation at 72 °C for 10 min. Negative extraction, qPCR, and positive (*Brassica oleracea*, cauliflower DNA) controls were also included in the reactions. The positive control was chosen as a species that displayed optimal amplification in laboratory workflows to provide a baseline comparison for other samples. Furthermore, this species was not anticipated to occur in any of our study sites, and therefore, any sources of cross-contamination from this positive would be easily recognised in the resulting sequences (Bohmann et al. 2022).

Following qPCR, dilutions that showed the optimal level of amplification (template amount relative to any inhibition) were amplified with ‘fusion primers’, which are gene-specific primers labelled on both the forward and reverse with 6–8 bp molecular identification (MID) tags coupled to Illumina sequencing adaptors. Each sample was tagged with a unique combination of forward and reverse MID tags not previously used within the laboratory, and qPCR reactions were prepared in an ultra-clean laboratory free from extracted or amplified DNA to minimise the possibility of contamination. Samples were amplified in duplicate using the qPCR conditions mentioned above to reduce the effects of PCR stochasticity (Murray et al. 2015). This included extraction and qPCR negative controls, but not qPCR positive controls. Using the qPCR results, PCR products were pooled in approximate equimolar concentration pools based on amplification curves, including negative controls. Pools were then quantified using a QIAxcel Advanced System (Qiagen) with the QIAxcel DNA High-Resolution Kit. As per the results of the quantification, sample pools were then combined in approximate equimolar ratios to create a sequencing library for each assay (*trn*L and ITS2). The *trn*L library was size-selected using a Pippin Prep 2% agarose Marker B cassette (Sage Science, USA) for fragments between 160 and 450 bp long, and the ITS2 library was size-selected for 200–650 bp on a Pippin Prep 1.5% Marker K cassette (Sage Science). Library pools were then purified using a QIAquick PCR purification kit (Qiagen) as per the manufacturer's instructions with the addition of a 5 min incubation at room temperature before elution. The purified library was eluted in 40 µl and quantified with a QuBit (Invitrogen, USA) using double-stranded DNA high-sensitivity reagents to determine the optimal volume of the library required for sequencing. Both libraries were sequenced on an Illumina MiSeq (Illumina, USA). The *trn*L libraries were sequenced on a single-end 300 cycle V2 kit, and the ITS2 libraries were sequenced on a paired-end 600 cycle V3 kit as per the manufacturer's directions.

### Bioinformatics and sequence processing

Unidirectional and unmerged paired-end sequencing reads were demultiplexed (assigned to their appropriate sample using the MID-tag combos) using 'Obitools' (Boyer et al. 2016) for the *trn*L dataset. To retain the paired-end data in the ITS2 dataset as unmerged reads for analysis using the ‘DADA2’ package (Callahan et al. 2016), demultiplexing was carried out using the default parameters in the 'insect' package (Wilkinson et al. 2018) in R v 3.6.1 (R Core Team 2019). Sequencing data were then quality filtered (*trn*L: minimum length = 50, maximum expected error = 2, no ambiguous nucleotides; ITS2: minimum length = 100, maximum expected error = 2, no ambiguous nucleotides), denoised, with paired-end reads (ITS2) merged with a minimum overlap length of 12, sequences identified as chimeras removed, and then dereplicated using the ‘DADA2’ package (Callahan et al. 2016) to produce Amplicon Sequence Variants (ASVs). ASVs were then curated using the ‘LULU’ package at default parameters (Frøslev et al. 2017). ASVs were matched to the NCBI GenBank reference database (www.ncbi.nlm.nih.gov/genbank/) using the Basic Local Alignment Search Tool (BLAST) for taxonomic assignment on a high-performance cluster computer (Pawsey Supercomputing Centre). BLAST results returned the top 10 hits with a minimum query coverage of 95% and a minimum percentage identity of 85%. These values were set based on of the poor availability of reference sequences in GenBank, and therefore improve likelihood of detection (Ryan et al. 2022; van der Heyde et al. 2020). Taxonomic assignments were made to the lowest common ancestor (LCA) using MEGAN [METAGenome Analyser v 6.13.5 (Huson 2018)] with a minimum score of 50 for *trn*L and 150 for ITS2. Plant taxa were cross-referenced to the Atlas of Living Australia (www.ala.org.au) and plant surveys of the sites (Prendergast and Ollerton 2021).

To determine the plant communities associated with the bee nesting tubes, only ASVs identified as plants (Phylum: Streptophyta) were retained in the analysis. ASV tables from both markers were then combined, retaining their ASV identity from each assay independent of taxonomy. Further filtering was then carried out on the entire data set. Any ASVs present in the negative control samples were removed using the ‘phyloseq’ package (McMurdie and Holmes 2013). Using the combined ASV table, a 0.05% minimum abundance filtering threshold was set within each sample to combat false, low abundance ASVs from each sample across with R 3.6.1 (R Core Team 2019). Minimum abundance filtering is equivalent to conducting rarefaction on the dataset without the need to remove low abundance samples (Prodan et al. 2020). Using ‘phyloseq’ (McMurdie and Holmes 2013), low occurrence ASVs with less than five sequences and occurring in only one sample were also removed. We removed any samples with less than 12,000 reads as this was where most samples had reached asymptote on a rarefaction curve (Fig S1).

### Statistical analysis

To establish the differences between the two different habitat types, a one-way PERMANOVA (fixed factor of ‘habitat’ with two levels: ‘residential garden’ and ‘bushland remnant’) was conducted on the normalised habitat characteristic values outlined above with Euclidian distance and 9999 permutations using the PERMANOVA + add on (Anderson et al. 2008) for PRIMER 7 (Clarke and Gorley 2015). A Principal Coordinate Analysis (PCO) based on Euclidian distance was performed on the normalised data from the measured habitat characteristics. The number of flowers and the number of native flowers were found to be co-linear variables (*r* = 0.96); however, because of the importance of these two characters for describing the habitat, they were retained for the analysis despite collinearity. The relative contribution of each habitat characteristic to the differences between habitat types was evaluated using the strength of the correlation coefficient to the PCO axes. Vectors were plotted to illustrate the strength and direction of the association.

Statistical analysis on sequencing data was performed using R 3.6.1 (R Core Team 2019) and PRIMER 7 (Clarke and Gorley 2015). The ASV abundance matrix was converted to presence–absence data, and all plant community statistics were calculated from this matrix. As a measure of alpha diversity, ASV richness was calculated using the DIVERSE function in PRIMER 7. A Euclidean distance resemblance matrix was made. ASV richness was tested using a univariate Permutational Analysis of Variance (PERMANOVA) with three factors, Habitat (fixed, two levels), Lecty (fixed, two levels), and Species, (random, nested within Lecty, varying levels). A Pearson correlation test was also conducted on the observed plant ASV richness and the number of nesting tubes using R 3.6.1 (R Core Team 2019).

The effect of Habitat, Lecty, and Species on the plant community composition was tested in the same way with a Permutational Multivariate Analysis of Variance (PERMANOVA) using Jaccard similarity with 9999 permutations using the PERMANOVA + add on (Anderson et al. 2008) for PRIMER 7 (Clarke and Gorley 2015). The multivariate dispersions around the centroid for habitat were tested for each of the bee species using the PERMDISP function in the PERMANOVA + add on (Anderson et al. 2008) for PRIMER 7 (Clarke and Gorley 2015). The plant community composition was illustrated with Non-metric Multi-dimensional Scaling (NMDS) using Jaccard Similarity with the 'vegan' package (Oksanen et al. 2019) and 'ggplot2' (Wickham 2016). Similarity percentage analysis (SIMPER) was used to identify plant families responsible for the differences between habitat type using PRIMER 7 (Clarke and Gorley 2015) based on the ASVs that could be identified to a family level. A distance-based linear model (DistLM) was used to characterise the relationship between the measured habitat characteristics and plant ASVs found in nesting tube contents. This model also included the factors Habitat, Lecty, and Species. The DistLM was done using the BEST selection procedure and the Akaike Information Criterion with correction (AICc) selection criterion using PERMANOVA + add on (Anderson et al. 2008) for PRIMER 7 (Clarke and Gorley 2015).

## Results

### Residential gardens and remnant bushland habitats

**Fig. 2:**
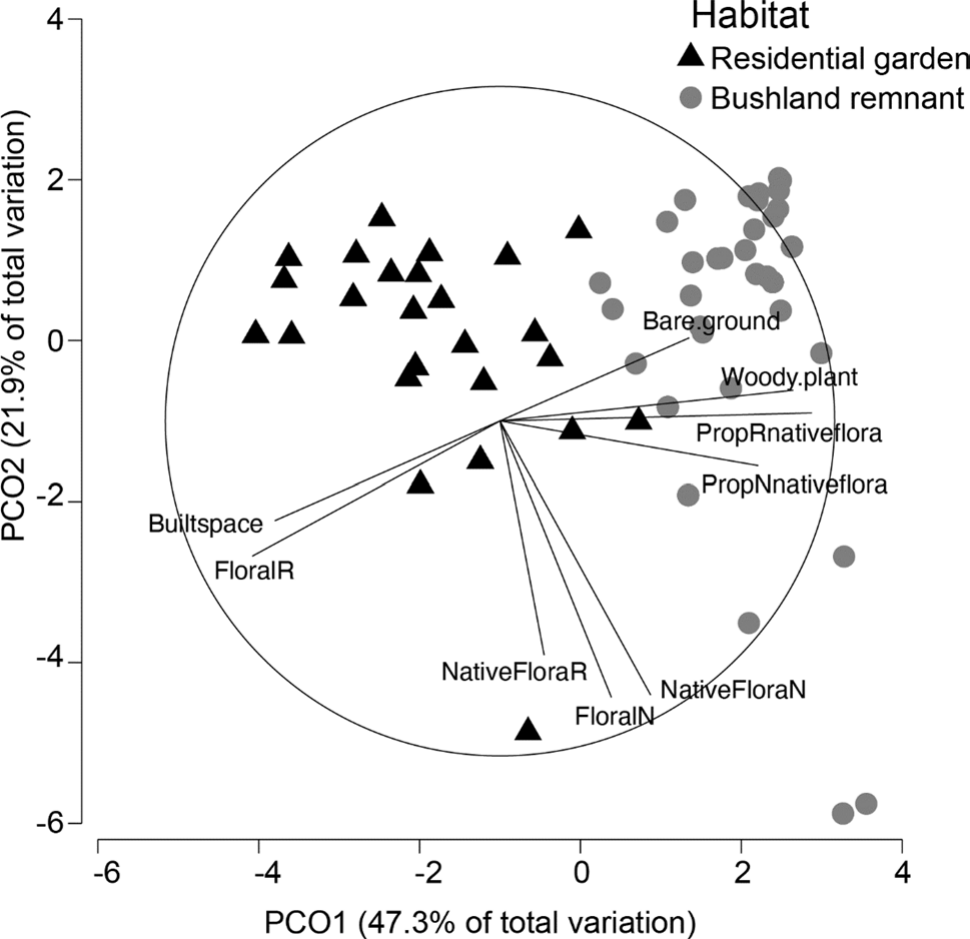
Principal Coordinates Analysis (PCO) plot of the measured habitat characteristics between bushland remnant (grey circle) and residential garden (black triangle) habitat types. The vectors plotted illustrate the strength and direction of the correlations of habitat characteristics to the PCO axes. For descriptions of abbreviations of habitat characteristics, see Supplementary Table 3

There was a significant difference between the habitat types (residential garden and bushland remnant) based on the measured habitat characteristics (PERMANOVA, *F*_(1,131)_ = 89.1, *p* = 0.001). PCO showed that 69.2% of the variation among the two habitat types was explained by axes 1 and 2 (Fig. 2). Residential gardens were associated with a greater percentage of built space and floral species richness, whilst remnant bushland was associated with the greater richness and abundance of native plant flowers and bee species, woody plants, and bare ground (Fig. 2, see also Prendergast et al. 2021).

### Sequencing results

The *trn*L and the ITS2 assays generated 8,949,032 (mean = 113,114 ± 812 SE sequences per sample) and 17,419,536 (mean = 72,646 ± 501 SE sequences per sample) quality filtered and ‘LULU’-curated sequences, respectively. Only six ASVs were detected within the negative control samples, two from the ITS2 assay and four from the *trn*L assay. As per Bell et al. (2017), these signified low levels of contamination either in the reagents or from sampling/laboratory workflows as the ASVs were from a subset of some of the most common taxa detected (Myrtaceae spp. and Fabaceae spp.). These ASVs were removed from further analysis. Analysis was then conducted on 14,521,974 sequences from 213 ASVs and 115 samples.

In total, there were 40 families, 50 genera, and 23 species of terrestrial vascular plants detected through metabarcoding of the bee nesting tubes. The majority of the metabarcoding detections belonged to the family Myrtaceae (103 ASVs), followed by Fabaceae (23 ASVs), Poaceae (10 ASVs), and Asteraceae (10 ASVs). There were several plant families detected through metabarcoding of the nesting tubes that were not observed as floral hosts in plant–pollinator surveys within the same geographic region (Table S1). These families include both native and exotic plant species (Fig S2, Table S3) that are either native to the area or can be found in residential gardens or road-side verges. Here, we define exotic plants as those that are exotic to Australia.

Both assays performed similarly at higher taxonomic levels; at least 99.1% of ITS2 ASVs and 88.7% of *trn*L ASVs were able to be identified to family level. This was markedly reduced at finer taxonomic levels for the *trn*L assay where only 43.9% of ASVs could be identified to genus level, whilst 97.4% of ITS2 ASVs could be identified to genus level; however, this was predominantly *Eucalyptus* ASV detections. At a species level, both assays performed similarly with 29.6% of the ITS2 ASVs identified to species and 23.5% of *trn*L ASVs identified to species. Even though the *trn*L assay had a limited taxonomic resolution, it detected a broader range of plant families (36) than the ITS2 assay (15), with 10 plant families shared between the two (Fig S2). For both ITS2 and *trn*L, there was a higher relative sequence abundance (from presence–absence data) from the Myrtaceae family than any other plant family within the dataset (Fig S3). However, whilst the ITS2 data were dominated by Myrtaceae sequences, the *trn*L dataset showed higher proportions of other families, such as Fabaceae (Fig S3).

### Native bee nest provision in residential gardens and urban bushland fragment habitats

**Fig. 3:**
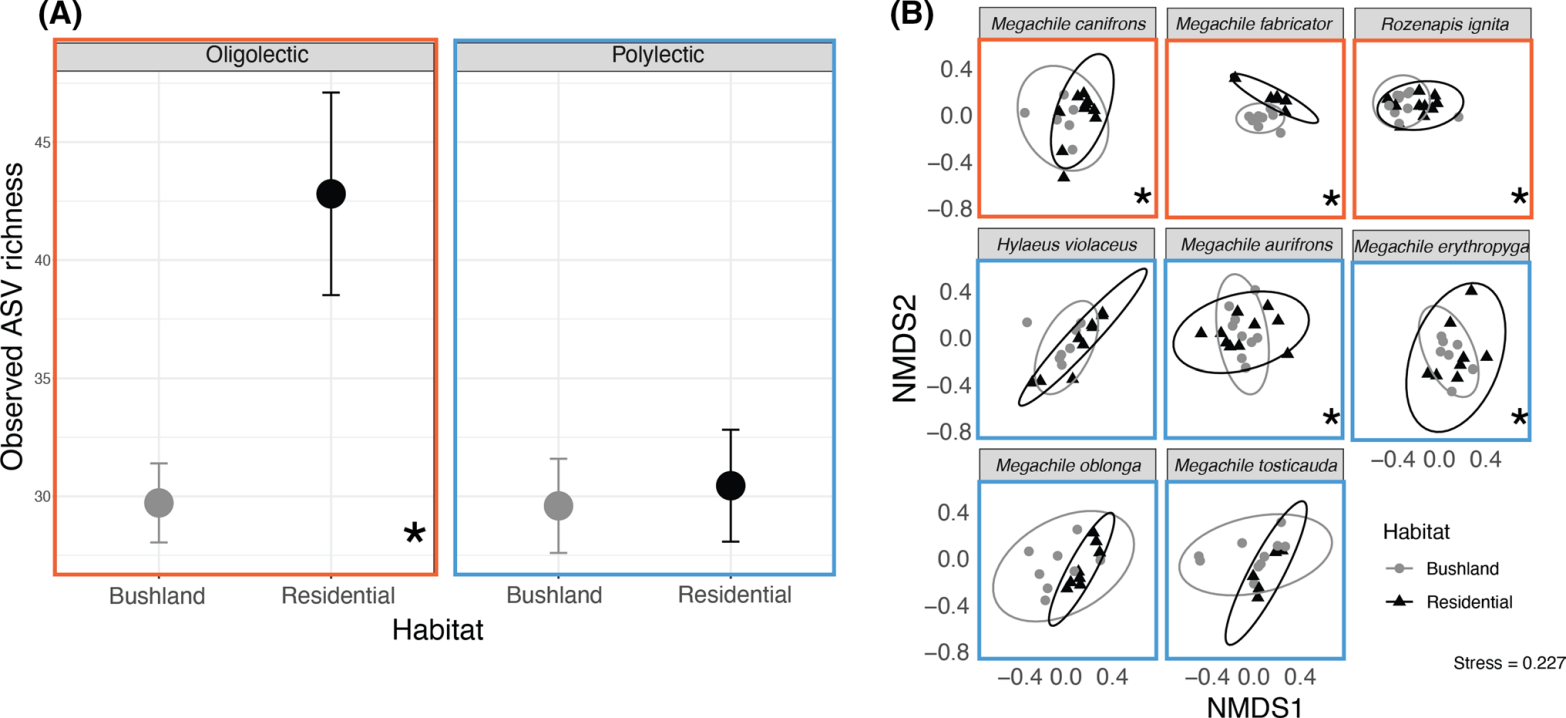
**A** Mean observed ASV richness (± S.E.) of plant taxa detected within nesting tubes of each species of beeoligolectic (specialists, orange box) and polylectic (generalists, blue box) bees for bushland remnant (grey circle) and residential garden (black circle) habitat type. Asterisk (*) indicates pairwise significant difference (*α* = 0.05). **B** Non-metric Multi-dimensional Scaling (NMDS) plot with Jaccard Similarity showing species of bees and the forage composition of nesting tubes between those in bushland remnant (grey circle) and residential garden (black triangle) habitat type. 95% Confidence intervals illustrated with circles corresponding to colour of bushland remnants (grey) and residential gardens (black). Species in the blue box are polylectic, whilst those in the orange box are oligolectic (specialists)

A univariate PERMANOVA on the observed ASV richness showed a significant interaction between habitat type and lecty, as well as a main effect of both habitat and lecty (Table 1). There was not a significant effect of bee species, or a significant interaction between habitat and species. Post hoc tests on the interaction of habitat and lecty identified that oligolectic (specialist) bees had greater ASV richness within their nest tubes in residential gardens than in Bushland (Table 1, Fig. 3A). However, there was no significant difference in the ASV richness in gardens or bushland for polylectic (generalist) bees (Table 1, Fig. 3A). Between the habitat types, residential gardens had a higher observed ASV richness (mean 35.3 ± 2.3 SE) than bushland habitat types (mean = 29.6 ± 1.4 SE, Table 1).

**Table 1:** Analysis of variance results for plant ASVs detected through metabarcoding. PERMANOVA main tests of ASV Richness and Plant ASV composition are presented

Term	DF	ASV Richness				Plant ASV composition			
		MS	*Pseudo-F*	*P* (perm)	Unique perms	MS	*Pseudo-F*	*P* (perm)	Unique perms
Habitat	1	1678	22.792	0.001*	9862	13,853	3.19	0.018*	9959
Lecty	1	1467	4.809	0.052	8538	7169	0.92	0.495	8815
Species (Lecty)	6	306	1.319	0.257	9938	7791	2.95	< 0.001*	9786
Habitat x Lecty	1	1508	20.482	0.001*	9864	4691	1.08	0.390	9956
Habitat x species (Lecty)	6	73	0.315	0.929	9957	4347	1.64	< 0.001*	9748
Residual	117	232				2645			

		Observed ASV richness Lecty x habitat			Plant ASV composition species x habitat		
Bee species	Lecty (*oligolectic *(specialist)* vs polylectic *(generalist))	*t *value	*P *value	*P *Adj	*t *value	*P *value	*P *Adj
Post hoc pairwise comparisons							
* Megachile canifrons*	Oligolectic	4.843	0.036*	0.072	1.324	0.039*	0.156
* Megachile fabricator*					1.904	< 0.001*	0.008*
* Rozenapis ignita*					1.769	< 0.001*	0.008*
* Hylaeus violaceus*	Polylectic	0.229	0.829	0.829	1.247	0.089	0.267
* Megachile aurifrons*					1.562	0.001*	0.008*
* Megachile erythropyga*					1.311	0.012*	0.060
* Megachile oblonga*					1.189	0.096	0.267
* Megachile tosticauda*					1.249	0.098	0.267

These are followed by post hoc pairwise comparisons for the significant interaction terms, Habitat x Lecty for ASV richness and Habitat x Species for Plant ASV composition. Habitat types are Residential Gardens or Remnant Bushland. *P* Adj values are adjusted with a post hoc Holm–Bonferroni correction due to multiple comparisons

*Indicates significance at *α* = 0.05

The PERMANOVA on the composition of plant ASVs in nesting tubes found that there was a significant interaction of bee species and habitat type (Table 1, Fig. 3B), and also differences between habitat type and bee species (Table 1). Further assessment of the interaction difference in the composition of plant taxa ASVs detected from residential garden and bushland nesting tubes for all the oligolectic species (*M. canifrons, M. fabricator,* and *R. ignita*) but only two of the polylectic species (*M. erythropyga* and *M. aurifrons*) (Fig. 3B, Table 1).

Analysis of multivariate dispersions (PERMDISP) indicated no significant difference in the diversity of forage between residential gardens and bushland remnants for most bee species (*p* > 0.05). The exception was the polylectic species *M. oblonga* (*F*_(1,15)_ = 6.562, *p* = 0.021), with forage ASVs in the Residential Gardens being less variable (mean dispersion 46.76 ± 1.68 SE) than in the Bushland Remnants (52.74 ± 1.61 SE).

SIMPER analysis indicated that Myrtaceae and Fabaceae were the most common detections in residential gardens and bushland remnant habitat types (Table S4). This was to be expected as Myrtaceae and Fabaceae were the most common plant families detected across the dataset (Fig S3). Results from the SIMPER analysis based on the plant families of detected ASVs indicated that there was an observed decrease in the frequency of Fabaceae ASVs contributing to the similarity within residential gardens (Table S4).

We found that although there were some habitat characteristics that had statistically significant relationships with the observed plant ASV composition, these variables could only explain a very low percentage of the variation in composition (Table S5). The overall BEST solution indicated that the three factors Habitat, Species, and Lecty together explained 17% of the variation in plant ASV composition. Additional variables that were included in alternative models within 2 AICc of the BEST model were related to the number of plant species or the number of native plant species (floral richness, native floral richness, and proportion of richness which is native flora) or distance to bushland.

## Discussion

Our study showed that eDNA metabarcoding can reveal the contents of nesting tubes, using eight native, cavity-nesting bee species in bushland remnants and residential gardens in Western Australia. Contrary to our hypothesis, oligolectic (specialist) bee species identified in our study (designations defined by Houston 2000 and Prendergast and Ollerton 2021) showed significantly higher species richness of plant hosts in residential gardens than in bushland remnant sites, and all our oligolectic species showed significantly different forage composition between habitat types. In comparison, for the majority of polylectic bee species, there was no significant difference between habitat types in richness or forage composition derived from the nesting tubes. This suggests a much more complex response of native bee species to urbanisation than previously thought.

### Urban survival and forage flexibility for oligolectic bee species

Contrary to our hypothesis, the oligolectic bee species had different ASV richness between habitat types, with higher richness observed in the residential gardens. There are several potential explanations for this. Higher diversity could indicate greater availability of forage in these habitat types for native bees, although, considering the co-evolution of native bees to their native host plants (Houston 2000; Phillips et al. 2010) and that these species are oligolectic, this seems unlikely. Instead, we suggest that this is an indicator of lower availability of preferred resources. This was supported by the increase in similarity of forage composition within residential gardens for these bees. As a result, we suggest that even these specialist native bees can expand their diet breadth to meet their resource requirements in suboptimal habitats. It should be noted that these oligolectic bee species were chosen as they were commonly found in our residential garden and remnant bushland study sites and therefore allowed us to achieve an adequate sampling size. As such, these species could be considered ‘urban adapters’ (McKinney 2002) in these spaces, as they have broad ecological adaptations that have positively translated in urban environments to allow them to forage and reproduce efficiently enough to allow populations to be maintained. One generalised adaptation is that the oligolectic bee species in our study could diversify their forage sources, indicating phenotypic plasticity (also known as behavioural flexibility). Behavioural flexibility is an important characteristic required for animals to be successful in urban environments (Lowry et al. 2013). This finding is supported by previous observations in these same residential garden sites, where native bees would visit native plants even if they were not native to the local area (Prendergast and Ollerton 2021). Phenotypic plasticity, generalisation, and dispersal ability have been identified as important characteristics required for survival in urban environments (Santini et al. 2019).

The oligolectic bee species in our study also had a generally larger intertegular span than did the polylectic species in this study, indicating that they can theoretically fly farther (Wright et al. 2015) to meet resource requirements. They may therefore be able to increase the diversity of their forage, as reflected by the contents of the nesting tubes. With residential gardens in our study characterised by higher floral richness and increased built space than bushland remnants, this could mean that these larger oligolectic bees were able to navigate through these spaces to find adequate forage resources. The habitats within our study were only surveyed within a 100 × 100 m area and bees have been documented to forage from 300 m to 1 km depending on their body size (Greenleaf et al. 2007). This is supported by previous research where bee communities in urbanised, fragmented vegetation were dominated by bee species with a greater flight range than in nature reserve areas (Hung et al. 2019). However, longer flight distance to forage for resources may reduce fitness of solitary bees by reducing their offspring production (Zurbuchen et al. 2010a) and lifespan due to the wear and stress posed on the exoskeleton and flight muscles (Torchio and Tepedino 1980). Whilst these oligolectic bees may survive in urban areas, there may be unknown physiological and reproductive consequences to living in urbanised areas that could impact the overall health of these bee populations.

In contrast, only two of our polylectic bee species showed no significant differences in the forage composition between habitat types. Although there are more exotic plant species in residential gardens than in bushland fragments, residential gardens were not devoid of native flowering plant species (Prendergast 2020). This suggests that the generalist bee species can access the same range of forage in residential gardens that they would in bushland remnants. These results reflect those of Buchholz et al. (2020) who found that urbanisation leads to an increase in the number of polylectic bee species. However, even though the polylectic bee species *M. oblonga* showed no significant difference in the ASV composition of its forage between habitat types, there was significantly smaller dispersion observed for the ASVs in the residential gardens than in the bushland habitats. This indicates reduced diversity of forage availability for this species in urban areas. Similarly, oligolectic bee species with significant differences in forage richness between habitat types also demonstrated a significant difference in the composition of forage in nesting tubes. A significant difference in composition could indicate that these bee species are able to access the varying resources—exotic or ornamental native plant species—available in urban environments, even if these foraging sources may not be preferred. As lecty is also considered through family-level specialisation, this might mean that oligolectic bee species are feeding from multiple species within a plant family. Furthermore, the distinction between urban and bushland environments in forage resources, especially for oligolectic bees, can suggest that these species are having to change their foraging behaviour to a higher degree than the polylectic species that showed no effect.

For both the oligolectic *M. canifrons* and *M. fabricator*, composition of forage resources in nesting tubes was characterised by *Eucalyptus* ASVs (family Myrtaceae), which is a common native genus and frequent in horticultural plantings (Prendergast and Ollerton 2021). Myrtaceae ASVs also contributed to a significant percentage of the similarity in residential gardens, potentially in the absence of preferred Fabaceae forage. Whilst *M. canifrons* and *M. fabricator* are Fabaceae specialists, lecty specialisation refers to pollen specialisation and not nectar (Cane and Sipes 2006); it may be that these additional plant taxa recorded in the specialist bees’ tubes represent DNA from nectar sources. One of the limitations of the current methods is that they cannot accurately quantify the relative proportions of plant species present, nor determine whether the sources were derived from nectar or pollen foraging. Furthermore, these detections could also represent resin gathered from *Eucalyptus* trees to create partitioning between brood cells in nesting tubes (Houston 2000). Additional research is required to determine the fitness consequences, if any, of how these differences in pollen diversity and composition in nesting tubes affect the native bee progeny (Filipiak and Filipiak 2020).

### DNA metabarcoding for taxonomic identification of plants

Prior studies have shown that DNA metabarcoding of pollen samples is simpler and provides greater taxonomic resolution than does traditional palynological approaches (Galimberti et al. 2014; Bell et al. 2017). However, this approach is not without limitations. Assays targeting shorter DNA fragments have been recommended for metabarcoding studies, because this DNA can be heavily degraded (Taberlet et al. 2007, 2012), but short fragments may lack the resolving power to discriminate at finer taxonomic levels (Pornon et al. 2016). The ITS2 region has been previously suggested as a useful region for molecular identification of eukaryotes, because it has fairly conserved regions across many taxonomic groups and contains a great deal of variability to distinguish closely related species (Chen et al. 2010; Yao et al. 2010). Nevertheless, both the assays used in our study showed limited species-level identification. This might also be explained by inadequate taxonomic representation in reference databases (Gous et al. 2021), which are limited for many floral taxonomic groups in Australia (Dormontt et al. 2018). Therefore, to compare the richness and composition of forage between bee species, we left ASVs independent of their taxonomy. This approach has been found to be an accurate proxy to estimate species diversity in the absence of adequate reference sequence databases (Ashfaq and Hebert, 2016; Gálvez-Reyes et al. 2020). However, taxonomic identification is still crucial for conservation, because species-level identification is important for effective conservation and management. These findings support the need for increased coverage of reference databases across a variety of Australian plant taxonomic groups to aid molecular taxonomic assignment (Bell et al. 2016).

The shorter *trn*L assay (~ 30–143 bp) detected a much wider range of plant families than did the ITS2 assay (~ 563 bp), which could suggest that larval digestion or other environmental factors may have degraded the eDNA and thus favour short amplicons. Dietary analysis using ITS2 plant assays is somewhat problematic as the amplicon length is too large to be reliably detected in degraded dietary items (Moorhouse-Gann et al. 2018). Further, interpretation of ITS2 data presents a challenge because of paralogous gene copies (Hollingsworth et al. 2011; Moorhouse-Gann et al. 2018), which may be a particular issue for eucalypts (Bayly et al. 2008). The ITS2 assay had very high numbers of *Eucalyptus* ASVs, which appeared to amplify in our samples preferentially. Thus, there needs to be a balance between taxonomic resolution and taxonomic breadth when choosing assays for metabarcoding studies. Therefore, we suggest a multi-assay approach, such as ours, to better distinguish plant communities from insect-gathered pollen (Pornon et al. 2016). Additionally, the ongoing development of group-specific (e.g., family) assays may help complement the use of assays such as ITS2 and *trn*L whose role is to provide a high-level assessment.

Whilst there are no known visual observations of any of the cavity-nesting bee species in our study foraging on members of Poaceae (Houston 2000; Prendergast 2020; Prendergast and Ollerton 2021), Poaceae ASVs were detected from 48 out of the 114 samples, equally among bee species and habitat types. In addition, a recent study that used metabarcoding of pollen from Australian native beehives similarly found unexpected detections of Poaceae (Wilson et al. 2021). Although Wilson et al. (2021) propose that these detections represent actual foraging activity, the results from the previous pollinator surveys at the sites in our study (Prendergast 2020; Prendergast and Ollerton 2021) do not support this hypothesis. Additionally, Poaceae constitutes a large proportion of total airborne pollen (Brennan et al. 2019), which suggests that Poaceae detections in our samples may instead have been airborne. Likewise, we cannot discern whether the detection of exotic plant ASVs from the nesting tubes represents actual foraging activity or background environmental accumulation. Previous observations from pollinator surveys showed no interactions between native bee species and exotic plants (Prendergast 2020; Prendergast and Ollerton 2021). Still, pollinator surveys undertaken through visual observation can be affected by bias based on observer, method, and context (O’Connor et al. 2019). Therefore, these exotic plant detections from nesting tubes represent directions for future research to explore the value of exotic plant species as a foraging resource for endemic native bees. For example, collection of data across different seasons to explore the persistence of the signal, and/or dissection of gastro-intestinal tracts directly from bees to avoid environmental background from the nesting tubes.

Our finding of a differential response of oligolectic and polylectic bee species to urbanisation adds to growing recognition that not all bees respond uniformly to ecosystem changes (Banaszak-Cibicka and Żmihorski 2012; Rader et al. 2014; De Palma et al. 2015). The bee species in our study were chosen as polylectic and oligolectic species that readily use urban environments, and these designations were defined through observation of their foraging behaviour in these environments (Prendergast and Ollerton 2021; Prendergast et al. 2021). The oligolectic bee species in our study demonstrated a shift from their preferred forage in bushland remnants to forage that was available in residential gardens. This same shift was not observed for polylectic species. Therefore, these species represent urban adapters in this region, with a degree of plasticity in their foraging preferences and resources. The shift in these resources currently has unknown future impacts for the health of bee species in urbanised areas. Previous work on cavity-nesting bees advocates for increasing the diversity of native forage available, especially in anthropogenically impacted landscapes (Gresty et al. 2018). However, there is little knowledge available on species-specific ecology and preferred host plants in urban environments for many Australian bee species, as most studies have been conducted in Europe or the Americas (Staab et al. 2018; Wenzel et al. 2020). Although studies on Australian native bees are increasing (Threlfall et al. 2015; Prendergast and Ollerton 2021; Prendergast et al. 2021), it is still imperative to continue research into the natural history of bee species here and around the globe.

The use of DNA metabarcoding can provide a valuable complement to dietary and other species-interaction studies through the ability to rapidly identify the composition of forage resources collected by bees and other organisms. As areas become more urbanised, future research on the impacts that changes in forage availability and composition have on the health and reproduction of fauna will be invaluable in conserving native fauna populations. For example, where metabarcoding was applied to the faecal material of songbirds, differences in diet in urban areas could be linked to decreases in offspring growth (Jarrett et al. 2020). Such research builds upon our knowledge of which species will be able to survive in urban environments, and which will completely avoid these areas or disappear from them. It is important that a more nuanced approach is taken to studying foraging preferences. For native bees, our results support the idea that lecty is a spectrum (Ritchie et al. 2016) and an individual species’ behavioural flexibility will have an influence on their survival in urban areas. Understanding the complexities of foraging behaviour in different organisms will be an important part of designing interventions to mitigate threats and build healthier urban ecosystems that can support high biodiversity.

## Supplementary Information

Below is the link to the electronic supplementary material.

Supplementary file1 (DOCX 516 KB)

## Data Availability

Sequencing data, bioinformatic scripts, and sample information can be found at: https://doi.org/10.5281/zenodo.5564128.
