# Supplementary material for: DNA metabarcoding identifies urban foraging patterns of oligolectic and polylectic cavity-nesting bees
Source: Oecologia. 2022 Sep 13;200(3-4):323–37. doi: 10.1007/s00442-022-05254-0 (PMC9675668; doi:10.1007/s00442-022-05254-0)

**Supplementary Materials:**

**DNA Metabarcoding identifies urban adaptation of oligolectic and polylectic cavity-nesting bees**

Kristen Fernandes*, Kit Prendergast, Philip W Bateman, Benjamin J Saunders, Mark Gibberd, Michael Bunce, Paul Nevill

* Corresponding Author: Kristen.fernandes@postgrad.curtin.edu.au

**Table S1:** Floral host and intertegular span of the eight species of bee included in this study, including number of nesting tubes sampled from each habitat type. Floral hosts were observed through pollinator surveys in both residential gardens and bushland remnant habitats (Prendergast and Ollerton 2021) and surveys by K.S. Prendergast (unpub) in southwest WA. Intertegular span was measured from female body size. Lecty is in terms of known specialisation on pollen in terms of foraging from flower species from a single family for oligolectic bees.

| **Species** | **Intertegular span (mm)** | **Lecty**  **(oligolectic (specialist) vs polylectic (generalist))** | **Floral host(s)** | **Residential garden** | **Bushland remnant** |
| --- | --- | --- | --- | --- | --- |
| *Megachile (Hackeriapis) canifrons* (Smith, 1853) | 3.17 | Oligolectic | *Jacksonia sternbergiana, J. furcellata,* and *Verticordia plumosa*† | 10 | 10 |
| *Megachile (Mitchellapis) fabricator* (Smith, 1868) | 3.57 | Oligolectic | *Jacksonia sternbergiana* and *J. furcellata* | 6 | 9 |
| *Rozenapis ignita* (Smith, 1853) | 3.25 | Oligolectic | *Jacksonia sternbergiana*, *J. furcellata,* and *Corymbia calophylla*† | 10 | 10 |
| *Hylaeus (Euprosopis) violaceus* (Smith, 1853) | 1.76 | Polylectic | *Melaleuca preissiana, M. huegelii, M. teretifolia*, *Eucalyptus spp.,* and *Banksia sessilis* | 9 | 8 |
| *Megachile aurifrons* (Smith, 1853) | 2.58 | Polylectic | *Scaevola spp.*, *Plectranthus spp.*, *Jacksonia furcellata*, *J. sternbergiana, Melaleuca quinquenervia,* and *Babingtonia camphorosmae* | 10 | 10 |
| *Megachile erythropyga* (Smith., 1853) | 3.2 | Polylectic | *Scaevola spp.*, *Plectranthus spp., Corymbia calophylla*, and *Callistemon spp.* | 10 | 10 |
| *Megachile (Hackeriapis) oblonga* (Smith, 1879) | 2.42 | Polylectic | *Corymbia callophylla, Goodenia filiformis, Jacksonia sericea, Melaleuca huegelii, Babingtonia camphorosmae, Cassytha racemosa, Stylidium brunonianum, Asartea scoparia, Baekea spp., Thryptomene saxicola,* and *Mentha pulegium* | 10 | 10 |
| *Megachile (Hackeriapis) tosticauda* (Cockerell, 1912) | 2.37 | Polylectic | *Babingtonia camphorosmae, Corymbia callophylla, Billardiera variifolia, Baekea spp.,* and *Cassytha racemosa* | 7 | 9 |

† Non-Fabaceae was nectar foraging given males were collected on these

**Table S2:** SIMPER analysis showing plant families ranked according to average Bray-Curtis dissimilarity between habitat types for overall native bee community and each species of bee. Frequency is based on abundance of ASVs identified to plant family found within those sites, a proxy for relative abundance.

| **Overall Bee Community** | | | |
| --- | --- | --- | --- |
| **Species** | **Frequency** | **Contribution%** | **Cumulative%** |
| **Bushland** | **Average Similarity: 60%** | | |
| Myrtaceae | 4.41 | 62.01 | 62.01 |
| Fabaceae | 2.1 | 24.5 | 86.51 |
| **Residential** | **Average Similarity: 57.8%** | | |
| Myrtaceae | 5.02 | 63.28 | 63.28 |
| Fabaceae | 2.02 | 22.22 | 85.5 |
| **Both** | **Average dissmilarity: 41.23%** | | |
| Myrtaceae | 4.41 | 21.06 | 21.06 |
| Fabaceae | 2.1 | 13.31 | 34.37 |
| Poaceae | 0.53 | 7.54 | 41.91 |
| Asteraceae | 0.43 | 6.17 | 48.09 |
| Proteaceae | 0.4 | 5.99 | 54.08 |
| Juglandaceae | 0.54 | 5.87 | 59.95 |
| Cupressaceae | 0.26 | 4.9 | 64.85 |
| Adoxaceae | 0.22 | 3.26 | 68.11 |
| Lauraceae | 0.17 | 2.89 | 71 |
| **Oligolectic Bees** | | | |
| ***Megachile canifrons*** | | | |
| **Species** | **Frequency** | **Contribution%** | **Cumulative%** |
| **Bushland** | **Average Similarity: 60.6%** | | |
| Myrtaceae | 4.23 | 63.56 | 63.56 |
| Fabaceae | 1.92 | 28.26 | 91.81 |
| **Residential** | **Average Similarity: 68.9%** | | |
| Myrtaceae | 6.69 | 72.06 | 72.06 |
| **Both** | **Average dissimilarity: 38%** | | |
| Myrtaceae | 4.23 | 30.19 | 30.19 |
| Fabaceae | 1.92 | 8.2 | 38.39 |
| Poaceae | 0.5 | 7.18 | 45.57 |
| Juglandaceae | 0.5 | 6.42 | 52 |
| Cupressaceae | 0.24 | 5.77 | 57.76 |
| Asteraceae | 0.4 | 5.71 | 63.47 |
| Proteaceae | 0.17 | 5.11 | 68.59 |
| Brassicaceae | 0.4 | 4.87 | 73.46 |
| ***Megachile fabricator*** | | | |
| **Species** | **Frequency** | **Contribution%** | **Cumulative%** |
| **Bushland** | **Average Similarity: 73.9%** | | |
| Myrtaceae | 5.21 | 66.78 | 66.78 |
| Fabaceae | 1.9 | 21.18 | 87.96 |
| **Residential** | **Average Similarity: 78.9%** | | |
| Myrtaceae | 7.29 | 72.96 | 72.96 |
| **Both** | **Average dissimilarity: 28.7%** | | |
| Myrtaceae | 5.21 | 33.03 | 33.03 |
| Fabaceae | 1.9 | 9.38 | 42.41 |
| Asteraceae | 0.11 | 7.56 | 49.97 |
| Loranthaceae | 0.11 | 6.82 | 56.79 |
| Poaceae | 0.33 | 6.21 | 63.01 |
| Proteaceae | 0.11 | 4.04 | 67.05 |
| Lauraceae | 0.22 | 3.24 | 70.29 |
| ***Rozenapis ignita*** | | | |
| **Species** | **Frequency** | **Contribution%** | **Cumulative%** |
| **Bushland** | **Average Similarity: 58.1%** | | |
| Fabaceae | 2.89 | 37.95 | 37.95 |
| Myrtaceae | 2.71 | 33.31 | 71.26 |
| **Residential** | **Average Similarity: 65.4%** | | |
| Myrtaceae | 4.03 | 37.81 | 37.81 |
| Fabaceae | 2.98 | 33.56 | 71.37 |
| **Both** | **Average dissmilarity: 39.2%** | | |
| Myrtaceae | 4.03 | 17.93 | 17.93 |
| Poaceae | 1.11 | 9.32 | 27.25 |
| Asteraceae | 0.44 | 8.82 | 36.08 |
| Fabaceae | 2.98 | 6.88 | 42.96 |
| Cupressaceae | 0.61 | 6.69 | 49.65 |
| Brassicaceae | 0.3 | 5.06 | 54.71 |
| Menyanthaceae | 0.3 | 4.77 | 59.48 |
| Juglandaceae | 0.5 | 4.56 | 64.04 |
| Rubiaceae | 0.3 | 4.21 | 68.25 |
| **Polylectic Bee Species** | | | |
| ***Hylaeus violaceus*** | | | |
| **Species** | **Frequency** | **Contribution%** | **Cumulative%** |
| **Bushland** | **Average Similarity: 61%** | | |
| Myrtaceae | 4.28 | 54.26 | 54.26 |
| Fabaceae | 2.29 | 26.63 | 80.89 |
| **Residential** | **Average Similarity: 56.6%** | | |
| Myrtaceae | 5.43 | 80.65 | 80.65 |
| **Both** | **Average dissimilarity: 39.2%** | | |
| Myrtaceae | 5.43 | 22.04 | 22.04 |
| Fabaceae | 1.42 | 16.88 | 38.92 |
| Poaceae | 0.74 | 8.28 | 47.2 |
| Juglandaceae | 0.29 | 7.43 | 54.63 |
| Cupressaceae | 0.29 | 5.52 | 60.15 |
| Proteaceae | 0.29 | 5.17 | 65.31 |
| Adoxaceae | 0.29 | 4.42 | 69.74 |
| Lauraceae | 0.29 | 4.27 | 74.01 |
| ***Megachile aurifrons*** | | | |
| **Species** | **Frequency** | **Contribution%** | **Cumulative%** |
| **Bushland** | **Average Similarity: 62%** | | |
| Myrtaceae | 4.2 | 63.15 | 63.15 |
| Fabaceae | 2.3 | 25.12 | 88.26 |
| **Residential** | **Average Similarity: 57.6%** | | |
| Myrtaceae | 4.14 | 48.72 | 48.72 |
| Fabaceae | 2.86 | 39.38 | 88.1 |
| **Both** | **Average dissimilarity: 41%** | | |
| Myrtaceae | 4.2 | 16.72 | 16.72 |
| Fabaceae | 2.3 | 11.98 | 28.7 |
| Asteraceae | 0.73 | 7.82 | 36.52 |
| Proteaceae | 0.6 | 7.52 | 44.04 |
| Malvaceae | 0 | 6.29 | 50.33 |
| Poaceae | 0.3 | 5.87 | 56.2 |
| Juglandaceae | 0.38 | 5.59 | 61.79 |
| Cupressaceae | 0.43 | 4.89 | 66.68 |
| Lythraceae | 0 | 3.71 | 70.39 |
| ***Megachile erythropyga*** | | | |
| **Species** | **Frequency** | **Contribution%** | **Cumulative%** |
| **Bushland** | **Average Similarity: 65.6%** | | |
| Myrtaceae | 5.09 | 69.25 | 69.25 |
| Fabaceae | 1.49 | 12.72 | 81.97 |
| **Residential** | **Average Similarity: 55.7%** | | |
| Myrtaceae | 4.32 | 58.54 | 58.54 |
| Cupressaceae | 1.17 | 16.87 | 75.41 |
| **Both** | **Average dissimilarity: 42.9%** | | |
| Myrtaceae | 4.32 | 13.19 | 13.19 |
| Fabaceae | 1.2 | 10.13 | 23.32 |
| Cupressaceae | 1.17 | 9.61 | 32.92 |
| Poaceae | 0.4 | 9.2 | 42.12 |
| Asteraceae | 0.63 | 7.23 | 49.35 |
| Juglandaceae | 0.6 | 5.38 | 54.73 |
| Verbenaceae | 0.6 | 5.35 | 60.08 |
| Solanaceae | 0.48 | 4.74 | 64.82 |
| Anacardiaceae | 0.4 | 4.1 | 68.91 |
| Podocarpaceae | 0.2 | 3.94 | 72.85 |
| ***Megachile oblonga*** | | | |
| **Species** | **Frequency** | **Contribution%** | **Cumulative%** |
| **Bushland** | **Average Similarity: 62.3%** | | |
| Myrtaceae | 4.94 | 65.83 | 65.83 |
| Fabaceae | 2.52 | 27.47 | 93.29 |
| **Residential** | **Average Similarity: 63.5%** | | |
| Myrtaceae | 4.57 | 69.64 | 69.64 |
| Fabaceae | 1.26 | 19.11 | 88.75 |
| **Both** | **Average dissimilarity: 38.4%** | | |
| Myrtaceae | 4.57 | 22.8 | 22.8 |
| Fabaceae | 1.26 | 21.8 | 44.6 |
| Poaceae | 0.34 | 8.92 | 53.52 |
| Juglandaceae | 0.71 | 7.65 | 61.17 |
| Asteraceae | 0.14 | 5.63 | 66.8 |
| Rubiaceae | 0.14 | 5.18 | 71.98 |
| ***Megachile tosticauda*** | | | |
| **Species** | **Frequency** | **Contribution%** | **Cumulative%** |
| **Bushland** | **Average Similarity: 66.4%** | | |
| Myrtaceae | 5.25 | 78.78 | 78.78 |
| **Residential** | **Average Similarity: 59%** | | |
| Myrtaceae | 4.93 | 67.3 | 67.3 |
| Fabaceae | 1.54 | 20.9 | 88.2 |
| **Both** | **Average dissimilarity: 34.4%** | | |
| Myrtaceae | 4.93 | 22.07 | 22.07 |
| Fabaceae | 1.54 | 13.04 | 35.11 |
| Adoxaceae | 0.5 | 7.76 | 42.87 |
| Poaceae | 0.25 | 7.7 | 50.57 |
| Juglandaceae | 0.75 | 6.79 | 57.36 |
| Asteraceae | 0.25 | 5.6 | 62.96 |
| Proteaceae | 0.25 | 5.45 | 68.41 |
| Lythraceae | 0.25 | 4.82 | 73.23 |

**Table S3:** Abbreviations used for habitat characteristics

| **Abbreviation** | **Description** |
| --- | --- |
| Area | Area of site |
| Builtspace | Percentage of built space within area |
| Dist.Bushland | Distance to nearest bushland |
| FloralN | Total number of flowers |
| FloralR | Species Richness of flowering plants |
| NativeFloraN | Number of native flowers |
| NativeFloraR | Species richness of native flowering plants |
| PropNnativeflora | Proportion of native flowers in total flowers |
| PropRnativeflora | Proportion of native flowering plant species richness in total species richness |
| Bare.ground | Bare ground cover |
| Woody.plant | Number of trees and shrubs |
| HoneybeeN | Number of honeybees observed |
| NativeBeeN | Number of native bees observed |
| TotalBeeN | Total number of bees observed |
| PropNativeBees | Proportion of native bees in total area |
| NativeBeeR | Native bee species richness caught during sampling period |

**Table S4:** List of plant detections from nesting tubes for bee species in this study

| **Oligolectic Bee Species** | | | | | | | | | |
| --- | --- | --- | --- | --- | --- | --- | --- | --- | --- |
| ***Megachile canifrons*** | |  | |  | |  | | |  |
| **Class** | **Order** | | **Family** | | ***Genus*** | | ***Species*** | | |
| Gunneridae | Myrtales | | Myrtaceae | | *Eucalyptus* | |  | | |
| Gunneridae | Myrtales | | Myrtaceae | |  | |  | | |
| Gunneridae | Asterales | | Asteraceae | |  | |  | | |
| Gunneridae | Myrtales | | Myrtaceae | | *Melaleuca* | | *Melaleuca nodosa* | | |
| Pinidae | Araucariales | | Podocarpaceae | | *Podocarpus* | |  | | |
| Pinidae | Cupressales | | Cupressaceae | | | |  | | |
| Gunneridae | Myrtales | | Lythraceae | | *Lagerstroemia* | | | | |
| Gunneridae | Brassicales | | Brassicaceae | | | |  | | |
| Gunneridae | Gentianales | | Rubiaceae | | *Galium* | |  | | |
| Gunneridae | Myrtales | | Myrtaceae | | *Ugni* | | *Ugni candollei* | | |
| eudicotyledons | Proteales | | Proteaceae | | *Persoonia* | |  | | |
| Gunneridae | Fabales | | Fabaceae | | *Jacksonia* | | *Jacksonia horrida* | | |
| Liliopsida | Poales | | Poaceae | |  | |  | | |
| Gunneridae | Fabales | | Fabaceae | |  | |  | | |
| Gunneridae | Dipsacales | | Adoxaceae | | *Viburnum* | |  | | |
| Gunneridae | Lamiales | |  | |  | |  | | |
| Gunneridae | Fagales | | Juglandaceae | | | |  | | |
| Magnoliidae | Laurales | | Lauraceae | |  | |  | | |
| Gunneridae | Fabales | | Fabaceae | | *Daviesia* | |  | | |
| Gunneridae | Pentapetalae | | | |  | |  | | |
| ***Megachile fabricator*** | |  | |  | |  | |  | |
| **Class** | **Order** | | **Family** | | **Genus** | | **Species** | | |
| Gunneridae | Myrtales | | Myrtaceae | | *Eucalyptus* | |  | | |
| Petrosaviidae | Poales | | Poaceae | | *Avena* | |  | | |
| Gunneridae | Myrtales | | Myrtaceae | |  | |  | | |
| Gunneridae | Asterales | | Asteraceae | |  | |  | | |
| Gunneridae | Fabales | | Fabaceae | |  | |  | | |
| Gunneridae | Myrtales | | Myrtaceae | | *Ugni* | | *Ugni candollei* | | |
| eudicotyledons | Proteales | | Proteaceae | | *Persoonia* | |  | | |
| Gunneridae | Fabales | | Fabaceae | | *Jacksonia* | | *Jacksonia horrida* | | |
| Gunneridae | Myrtales | | Myrtaceae | | *Melaleuca* | | *Melaleuca nodosa* | | |
| Gunneridae | Santalales | | Loranthaceae | | *Nuytsia* | | *Nuytsia floribunda* | | |
| Gunneridae | Dipsacales | | Adoxaceae | | *Viburnum* | |  | | |
| Gunneridae | Fagales | | Juglandaceae | | | |  | | |
| Magnoliidae | Laurales | | Lauraceae | |  | |  | | |
| Gunneridae | Fabales | | Fabaceae | | *Daviesia* | |  | | |
| Gunneridae | Pentapetalae | | | |  | |  | | |
| Liliopsida | Poales | | Poaceae | |  | |  | | |
| ***Rozenapis ignita*** | |  | |  | |  | | |  |
| **Class** | **Order** | | **Family** | | **Genus** | | **Species** | | |
| Gunneridae | Myrtales | | Myrtaceae | | *Eucalyptus* | |  | | |
| Pinidae | Cupressales | | Cupressaceae | | *Juniperus* | |  | | |
| Mesangiospermae | | |  | |  | |  | | |
| Gunneridae | Myrtales | | Myrtaceae | |  | |  | | |
| Gunneridae | Asterales | | Asteraceae | |  | |  | | |
| Gunneridae | Asterales | | Menyanthaceae | | | |  | | |
| Pinidae | Araucariales | | Podocarpaceae | | *Podocarpus* | |  | | |
| Pinidae | Cupressales | | Cupressaceae | | | |  | | |
| Gunneridae | Fabales | | Fabaceae | |  | |  | | |
| Gunneridae | Fabales | | Fabaceae | | *Hardenbergia* | | | | |
| Gunneridae | Brassicales | | Brassicaceae | | | |  | | |
| Gunneridae | Fabales | | Fabaceae | | *Jacksonia* | | *Jacksonia horrida* | | |
| Liliopsida | Poales | | Poaceae | | *Avena* | |  | | |
| Gunneridae | Fabales | | Fabaceae | | *Daviesia* | |  | | |
| Gunneridae | Solanales | | Solanaceae | |  | |  | | |
| Gunneridae | Gentianales | | Rubiaceae | | *Galium* | |  | | |
| Gunneridae | Myrtales | | Myrtaceae | | *Ugni* | | *Ugni candollei* | | |
| Liliopsida | Poales | | Poaceae | |  | |  | | |
| eudicotyledons | Proteales | | Proteaceae | | *Persoonia* | |  | | |
| Gunneridae | Sapindales | | Anacardiaceae | | *Schinus* | | *Schinus terebinthifolia* | | |
| eudicotyledons | Proteales | | Proteaceae | | *Grevillea* | |  | | |
| Gunneridae | Fabales | | Fabaceae | | *Lotus* | | *Lotus unifoliolatus* | | |
| Gunneridae | Apiales | | Apiaceae | |  | |  | | |
| Pinidae | Cupressales | | Cupressaceae | | *Callitris* | |  | | |
| Cycadidae | Cycadales | | Zamiaceae | | *Macrozamia* | | | | |
| Liliopsida | Poales | | Poaceae | | *Ehrharta* | |  | | |
| Gunneridae | Myrtales | | Myrtaceae | | *Melaleuca* | | *Melaleuca nodosa* | | |
| Liliopsida | Zingiberales | | Musaceae | |  | |  | | |
| Gunneridae | Dipsacales | | Adoxaceae | | *Viburnum* | |  | | |
| Gunneridae | Asterales | |  | |  | |  | | |
| Gunneridae | Lamiales | |  | |  | |  | | |
| Gunneridae | Fagales | | Juglandaceae | | | |  | | |
| Magnoliidae | Laurales | | Lauraceae | |  | |  | | |
| Gunneridae | Lamiales | | Oleaceae | | *Ligustrum* | |  | | |
| Gunneridae | Pentapetalae | | | |  | |  | | |
| **Polylectic Bee Species** | | | | | | | | | |
| ***Hylaeus violaceus*** | |  | |  | |  | | |  |
| **Class** | **Order** | | **Family** | | **Genus** | | **Species** | | |
| Gunneridae | Myrtales | | Myrtaceae | | *Eucalyptus* | |  | | |
| Gunneridae | Myrtales | | Myrtaceae | |  | |  | | |
| Gunneridae | Asterales | | Asteraceae | |  | |  | | |
| Pinidae | Araucariales | | Podocarpaceae | | *Podocarpus* | |  | | |
| Pinidae | Cupressales | | Cupressaceae | | | |  | | |
| Gunneridae | Fabales | | Fabaceae | |  | |  | | |
| Gunneridae | Brassicales | | Brassicaceae | | | |  | | |
| Gunneridae | Gentianales | | Rubiaceae | | *Galium* | |  | | |
| Gunneridae | Myrtales | | Myrtaceae | | *Ugni* | | *Ugni candollei* | | |
| Gunneridae | Fabales | | Fabaceae | | *Grazielodendron* | | | | |
| Liliopsida | Poales | | Poaceae | |  | |  | | |
| eudicotyledons | Proteales | | Proteaceae | | *Persoonia* | |  | | |
| Gunneridae | Fabales | | Fabaceae | | *Jacksonia* | | *Jacksonia horrida* | | |
| eudicotyledons | Proteales | | Proteaceae | | *Grevillea* | |  | | |
| Gunneridae | Myrtales | | Myrtaceae | | *Melaleuca* | | *Melaleuca nodosa* | | |
| Gunneridae | Dipsacales | | Adoxaceae | | *Viburnum* | |  | | |
| Gunneridae | Lamiales | |  | |  | |  | | |
| Gunneridae | Fagales | | Juglandaceae | | | |  | | |
| Magnoliidae | Laurales | | Lauraceae | |  | |  | | |
| Gunneridae | Pentapetalae | | | |  | |  | | |
| Pinidae | Araucariales | | | |  | |  | | |
| ***Megachile aurifrons*** | |  | |  | |  | | |  |
| **Class** | **Order** | | **Family** | | **Genus** | | **Species** | | |
| Gunneridae | Myrtales | | Myrtaceae | | *Eucalyptus* | |  | | |
| Gunneridae | Malvales | | Malvaceae | | *Talipariti* | | *Talipariti tiliaceum* | | |
| Gunneridae | Asterales | | Asteraceae | | *Cotula* | | *Cotula australis* | | |
| Gunneridae | Myrtales | | Myrtaceae | |  | |  | | |
| Gunneridae | Asterales | | Asteraceae | |  | |  | | |
| Gunneridae | Asterales | | Menyanthaceae | | | |  | | |
| Pinidae | Cupressales | | Cupressaceae | | | |  | | |
| Gunneridae | Fabales | | Fabaceae | |  | |  | | |
| Gunneridae | Myrtales | | Lythraceae | | *Lagerstroemia* | | | | |
| Gunneridae | Fabales | | Fabaceae | | *Hardenbergia* | | | | |
| Gunneridae | Fabales | | Fabaceae | | *Jacksonia* | | *Jacksonia horrida* | | |
| Gunneridae | Solanales | | Solanaceae | |  | |  | | |
| Gunneridae | Gentianales | | Rubiaceae | | *Galium* | |  | | |
| Gunneridae | Myrtales | | Myrtaceae | | *Ugni* | | *Ugni candollei* | | |
| Gunneridae | Fabales | | Fabaceae | | *Grazielodendron* | | | | |
| eudicotyledons | Proteales | | Proteaceae | | *Persoonia* | |  | | |
| eudicotyledons | Proteales | | Proteaceae | | *Grevillea* | |  | | |
| Gunneridae | Apiales | | Apiaceae | |  | |  | | |
| Gunneridae | Lamiales | | Verbenaceae | | | |  | | |
| Gunneridae | Myrtales | | Myrtaceae | | *Melaleuca* | | *Melaleuca nodosa* | | |
| Gunneridae | Brassicales | | Brassicaceae | | | |  | | |
| Gunneridae | Malvales | | Malvaceae | | *Talipariti* | |  | | |
| Gunneridae | Lamiales | |  | |  | |  | | |
| Gunneridae | Fagales | | Juglandaceae | | | |  | | |
| Magnoliidae | Laurales | | Lauraceae | |  | |  | | |
| Gunneridae | Lamiales | | Oleaceae | | *Ligustrum* | |  | | |
| Gunneridae | Fabales | | Fabaceae | | *Daviesia* | |  | | |
| Gunneridae | Pentapetalae | | | |  | |  | | |
| Liliopsida | Poales | | Poaceae | |  | |  | | |
| Gunneridae | Solanales | | Convolvulaceae | | | |  | | |
| ***Megachile erythropyga*** | |  | |  | |  | | |  |
| **Class** | **Order** | | **Family** | | **Genus** | | **Species** | | |
| Gunneridae | Myrtales | | Myrtaceae | | *Eucalyptus* | |  | | |
| Gunneridae | Myrtales | | Myrtaceae | | *Syzygium* | |  | | |
| Gunneridae | Myrtales | | Myrtaceae | |  | |  | | |
| Gunneridae | Asterales | | Asteraceae | |  | |  | | |
| Pinidae | Araucariales | | Podocarpaceae | | *Podocarpus* | |  | | |
| Pinidae | Cupressales | | Cupressaceae | | | |  | | |
| Gunneridae | Fabales | | Fabaceae | |  | |  | | |
| Liliopsida | Poales | | Poaceae | | *Avena* | |  | | |
| Gunneridae | Rosales | | Moraceae | | *Morus* | |  | | |
| Gunneridae | Solanales | | Solanaceae | |  | |  | | |
| Gunneridae | Myrtales | | Myrtaceae | | *Ugni* | | *Ugni candollei* | | |
| eudicotyledons | Proteales | | Proteaceae | | *Persoonia* | |  | | |
| Gunneridae | Sapindales | | Anacardiaceae | | *Schinus* | | *Schinus terebinthifolia* | | |
| Gunneridae | Gentianales | | Apocynaceae | | | |  | | |
| Gunneridae | Lamiales | | Verbenaceae | | | |  | | |
| Gunneridae | Fabales | | Fabaceae | | *Jacksonia* | | *Jacksonia horrida* | | |
| Liliopsida | Poales | | Poaceae | |  | |  | | |
| Cycadidae | Cycadales | | Zamiaceae | | *Macrozamia* | | | | |
| Gunneridae | Myrtales | | Myrtaceae | | *Melaleuca* | | *Melaleuca nodosa* | | |
| Gunneridae | Lamiales | |  | |  | |  | | |
| Gunneridae | Fagales | | Juglandaceae | | | |  | | |
| Gunneridae | Pentapetalae | | | |  | |  | | |
| Gunneridae | Solanales | | Convolvulaceae | | | |  | | |
| ***Megachile oblonga*** | |  | |  | |  | | |  |
| **Class** | **Order** | | **Family** | | **Genus** | | **Species** | | |
| Gunneridae | Myrtales | | Myrtaceae | | *Eucalyptus* | |  | | |
| Gunneridae | Myrtales | | Myrtaceae | |  | |  | | |
| Gunneridae | Asterales | | Asteraceae | |  | |  | | |
| Gunneridae | Asterales | | Menyanthaceae | | | |  | | |
| Gunneridae | Fabales | | Fabaceae | |  | |  | | |
| Gunneridae | Fabales | | Fabaceae | | *Jacksonia* | | *Jacksonia horrida* | | |
| Gunneridae | Gentianales | | Rubiaceae | | *Galium* | |  | | |
| Gunneridae | Myrtales | | Myrtaceae | | *Ugni* | | *Ugni candollei* | | |
| Gunneridae | Fabales | | Fabaceae | | *Grazielodendron* | | | | |
| eudicotyledons | Proteales | | Proteaceae | | *Persoonia* | |  | | |
| Gunneridae | Fabales | | Fabaceae | | *Lotus* | | *Lotus unifoliolatus* | | |
| Gunneridae | Myrtales | | Myrtaceae | | *Melaleuca* | | *Melaleuca nodosa* | | |
| Gunneridae | Santalales | | Loranthaceae | | *Nuytsia* | | *Nuytsia floribunda* | | |
| Gunneridae | Dipsacales | | Adoxaceae | | *Viburnum* | |  | | |
| Gunneridae | Asterales | |  | |  | |  | | |
| Liliopsida | Poales | | Poaceae | |  | |  | | |
| Gunneridae | Lamiales | |  | |  | |  | | |
| Gunneridae | Fagales | | Juglandaceae | | | |  | | |
| Gunneridae | Fabales | | Fabaceae | | *Daviesia* | |  | | |
| Gunneridae | Pentapetalae | | | |  | |  | | |
| ***Megachile tosticauda*** | |  | |  | |  | | |  |
| **Class** | **Order** | | **Family** | | **Genus** | | **Species** | | |
| Gunneridae | Myrtales | | Myrtaceae | | *Eucalyptus* | |  | | |
| Petrosaviidae | Poales | | Poaceae | | *Avena* | |  | | |
| Gunneridae | Myrtales | | Myrtaceae | |  | |  | | |
| Gunneridae | Fabales | | Fabaceae | |  | |  | | |
| Gunneridae | Myrtales | | Myrtaceae | | *Ugni* | | *Ugni candollei* | | |
| Gunneridae | Myrtales | | Myrtaceae | | *Melaleuca* | | *Melaleuca nodosa* | | |
| eudicotyledons | Proteales | | Proteaceae | | *Persoonia* | |  | | |
| Gunneridae | Fabales | | Fabaceae | | *Jacksonia* | | *Jacksonia horrida* | | |
| Gunneridae | Dipsacales | | Adoxaceae | | *Viburnum* | |  | | |
| Gunneridae | Lamiales | |  | |  | |  | | |
| Gunneridae | Fagales | | Juglandaceae | | | |  | | |
| Magnoliidae | Laurales | | Lauraceae | |  | |  | | |
| Gunneridae | Pentapetalae | | | |  | |  | | |
| Gunneridae | Solanales | | Convolvulaceae | | | |  | | |

**Table S5:** Distance based linear model (DistLM) analysis of plant ASV composition from brooding tubes across all sites. Constructed using a BEST selection procedure and the AICc selection criterion. The overall best solution is presented, and possible alternate models within 2 AICc of the best. * indicates significance at α = 0.05.

|  | Selections | Number of variables | AICc | R^2^ |
| --- | --- | --- | --- | --- |
| **Overall BEST solution** | Habitat. Species, Lecty | 3 | 1063.1 | 0.166 |
| **Alternate solutions within 2 AICc of BEST solution** | Habitat, Species | 2 | 1063.1 | 0.166 |
|  | Habitat. Species, Lecty, NativeFloraR | 4 | 1063.3 | 0.180 |
|  | Habitat. Species, Lecty, Floral Richness, PropRnativeFlora | 5 | 1063.8 | 0.192 |
|  | Habitat. Species, Lecty, Dist Bushland, Floral Richness, PropRnativeFlora | 6 | 1064.3 | 0.203 |
|  |  |  |  |  |
| **Marginal tests for each variable** | |  |  |  |
| Variable | Characteristic | Pseudo-F | Proportion | P value |
| 1 | Habitat | 4.214 | 0.031 | 0.0001* |
| 2 | Species | 2.784 | 0.135 | 0.0001* |
| 3 | Lecty | 2.37 | 0.017 | 0.0022* |
| 4 | Area | 2.848 | 0.021 | 0.0002* |
| 5 | Builtspace | 3.163 | 0.023 | 0.0001* |
| 6 | Dist.Bushland | 2.633 | 0.019 | 0.0005* |
| 7 | FloralN | 1.05 | 0.007 | 0.3615 |
| 8 | FloralR | 2.829 | 0.02 | 0.0002* |
| 9 | NativeFloraN | 1.082 | 0.008 | 0.3249 |
| 10 | NativeFloraR | 2.515 | 0.018 | 0.0008* |
| 11 | PropNnativeflora | 1.13 | 0.008 | 0.2676 |
| 12 | PropRnativeflora | 2.466 | 0.018 | 0.0014* |
| 13 | Bare.ground | 2.249 | 0.017 | 0.0046* |
| 14 | Woody.plant | 3.449 | 0.026 | 0.0001* |

**Supplementary Figures**

**Figure S1:** Rarefaction curve of raw reads (Sample Size) versus ASV richness (Species)


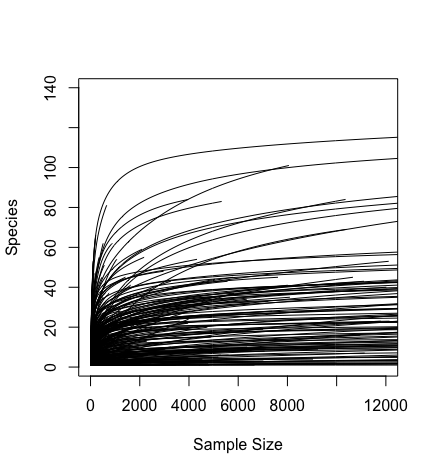


**Figure S2:** Cladograms of trees generated from taxa assignments to plant Family level for *trn*L and ITS2 primers. Green indicates shared taxa between the two primer sets.


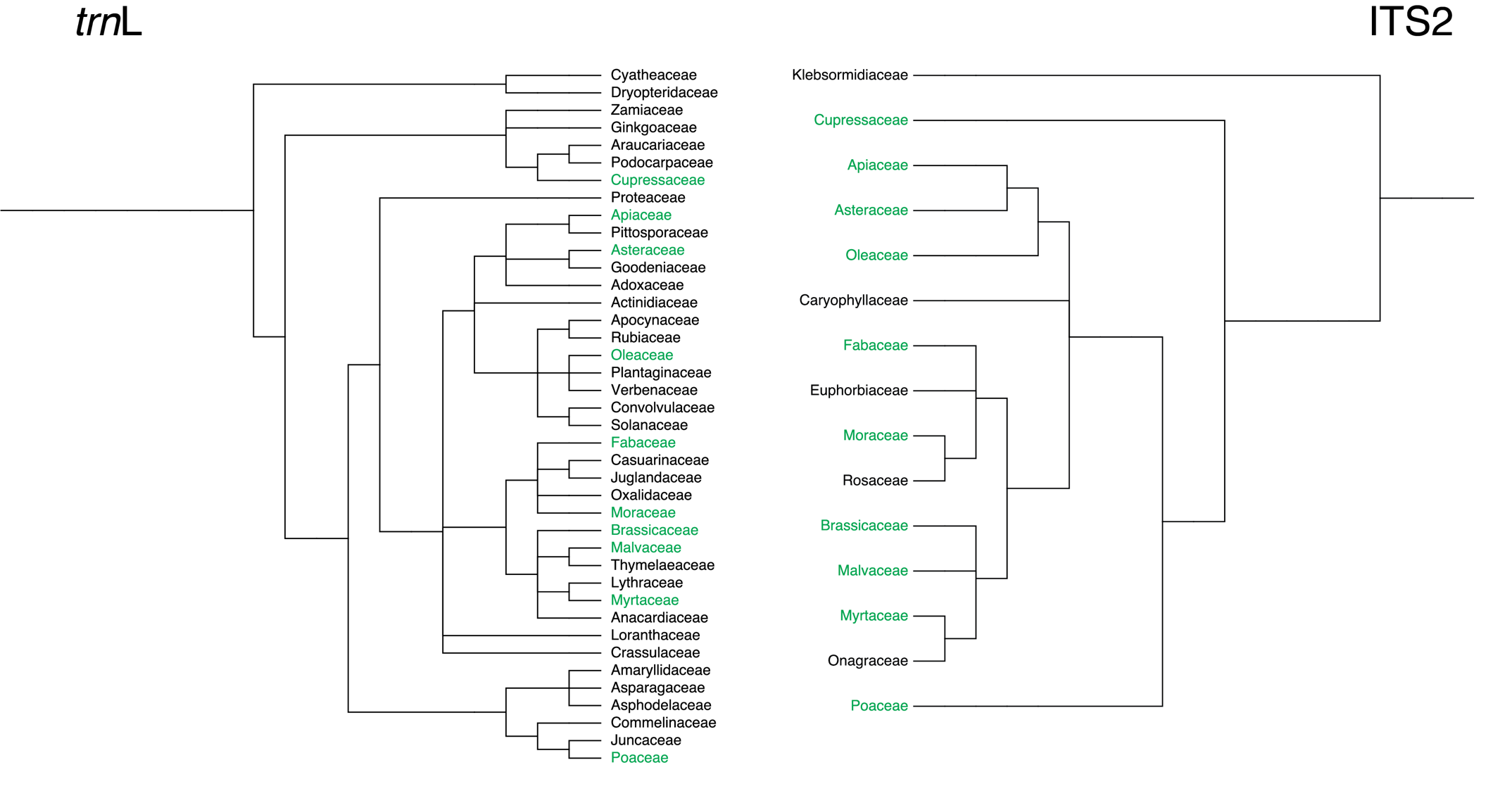


**Figure S3:** Relative sequence abundances (from presence-absence detections) of the two PCR assays (ITS2 and *trn*L) from all samples showing the proportions of ASVs that could be identified to Family level between Bushland Remnants and Residential Garden habitat types.

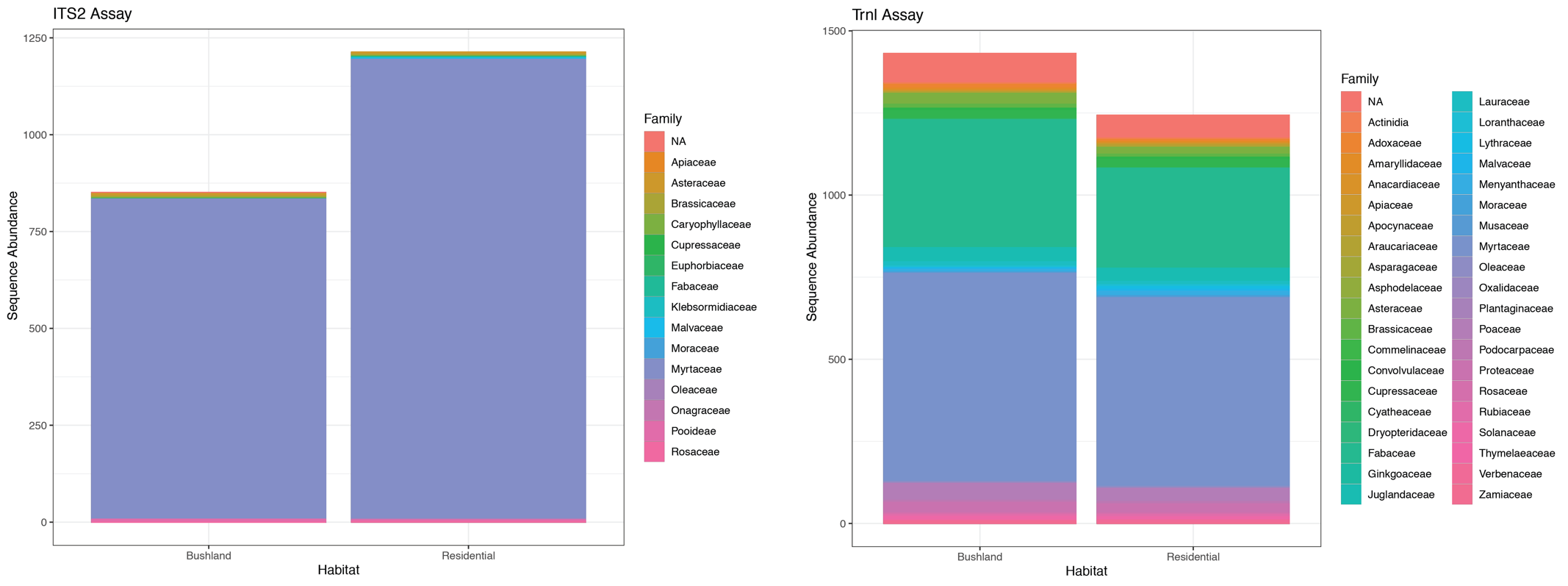

Supplement: Supplementary file 1 — Supplementary file1 (DOCX 516 KB) [file 442_2022_5254_MOESM1_ESM.docx]
